# Impaired binding of standard initiation factors eIF3b, eIF4G and eIF4B to domain V of the live-attenuated coxsackievirus B3 *Sabin3-like* IRES - alternatives for 5′UTR-related cardiovirulence mechanisms

**DOI:** 10.1186/1746-1596-8-161

**Published:** 2013-09-24

**Authors:** Amira Souii, Jawhar Gharbi, Manel Ben M’hadheb-Gharbi

**Affiliations:** 1 Institut Supérieur de Biotechnologie de Monastir, Université de Monastir, Avenue Tahar Hadded, BP 74, Monastir 5000, Tunisia

**Keywords:** Attenuation, CVB3, Domain V, IRES, eIFs, Translation initiation, UV-crosslinking, Filter-binding

## Abstract

**Abstract:**

Internal ribosome entry site (IRES) elements fold into highly organized conserved secondary and probably tertiary structures that guide the ribosome to an internal site of the RNA at the IRES 3′end. The composition of the cellular proteome is under the control of multiple processes, one of the most important being translation initiation. In each poliovirus Sabin vaccine strain, a single point mutation in the IRES secondary-structure domain V is a major determinant of neurovirulence and translation attenuation. Here we are extrapolating poliovirus findings to a genomic related virus named coxsackievirus B3 CVB3); a causative agent of viral myocarditis. We have previously reported that *Sabin3-like* mutation (U^473^ → C) introduced in the domain V sequence of the CVB3 IRES led to a defective mutant with a serious reduction in translation efficiency and ribosomal initiation complex assembly, besides an impaired RNA-protein binding pattern. With the aim to identify proteins interacting with both CVB3 wild-type and *Sabin3-like* domain V RNAs and to assess the effect of the *Sabin3-like* mutation on these potential interactions, we have used a proteomic approach. This procedure allowed the identification of three RNA-binding proteins interacting with the domain V: eIF4G (p220), eIF3b (p116) and eIF4B (p80). Moreover, we report that this single-nucleotide exchange impairs the interaction pattern and the binding affinity of these standard translation initiation factors within the IRES domain V of the mutant strain. Taken together, these data indicate how this decisive *Sabin3-like* mutation mediates viral translation attenuation; playing a key role in the understanding of the cardiovirulence attenuation within this construct. Hence, these data provide further evidence for the crucial role of RNA structure for the IRES activity, and reinforce the idea of a distribution of function between the different IRES structural domains.

**Virtual slide:**

The virtual slide(s) for this article can be found here: http://www.diagnosticpathology.diagnomx.eu/vs/6160165131045880.

## Background

Internal Ribosome Entry Site (IRES) elements are specialized RNA regulatory sequences governing cap-independent translation initiation in eukaryotic mRNAs that are translated during cellular stress, that is, when cap-dependent translation is compromised [1-4]. IRES elements, initially reported in the genomic RNA of two picornaviruses (namely, poliovirus (PV) and encephalomyocarditis virus (EMCV)), drive internal initiation of translation in the mRNA of all members of the *Picornaviridae* family [5-8]. Soon after their discovery, IRES elements were also found in other RNA viruses infecting mammals, such as hepatitis C (HCV), pestiviruses [9,10], or retroviruses [11-13], as well as in RNA viruses infecting invertebrates [14,15], plants [16], and protozoa [17]. Recently, IRES-dependent translation in mRNAs transcribed from DNA viruses belonging to the *Herpesviridae* family has been reported [18].

Initiation of translation in eukaryotes, which is the rate-limiting step in protein synthesis, involves a set of specialized proteins that recruit the small ribosome subunit to the m^7^GpppN structure (termed cap) located at the 5′-end of most mRNAs [19,20]. The cap-dependent initiation complex is thought to scan the 5′ untranslated regions (UTR) until an AUG codon is placed in the appropriate context to start protein synthesis [20-22]. In marked contrast with this mechanism, a few viral RNA initiate translation internally *via* the IRES using a cap-independent mechanism, bypassing proteins bound to the 5′ UTR as well as strong RNA structures in front of the start site [21].

Because IRESs differ in nucleotide sequence, RNA secondary structure and trans-acting factors requirement [23-25], deciphering the role of evolutionary conserved motifs is critical to understand internal initiation mechanisms. Functional and structural analysis of viral IRESs has shown that RNA structure plays a fundamental role for IRES-dependent translation initiation [26-28]. Consistent with this, compensatory substitutions tend to conserve RNA structure during RNA virus evolution [1,29]. Besides the structural organization, the IRES function relies on the interaction with cellular proteins [21]. Indeed, RNA-binding proteins play a crucial role in gene expression control in all organisms. In eukaryotic cells, a large variety of ribonucleoprotein complexes affect the processing, transport, localization, translation and decay of mRNAs [30,31]. Thus, RNA-binding proteins are responsible for the establishment and regulation of RNA–protein networks that determine the target mRNA fate. The untranslated regions of mRNAs play a key role in many of these processes, serving as platforms for the assembly of macromolecular complexes particularly those controlling translation initiation [20].

Myocarditis or inflammatory cardiomyopathy is inflammation of heart muscle which can be caused by several agents. Myocardial fibrosis occurs in a number of pathological processes, most commonly hypertension. Other disease states capable of producing cardiac fibrosis include hypereosinophilia, scleroderma, viral myocarditis and inherited genetic mutations [32]. In the same context, Lakhan and Harle [32] reported the case of an elderly, non hypertensive athlete who died suddenly of sepsis. Autopsy demonstrated foci of fibrosis throughout the right and left ventricle and significant narrowing of the left ventricular cavity.

The detection of viral genomes in endomyocardial biopsies by molecular techniques has greatly expanded the list of viruses implicated in myocarditis. Initially, the molecular diagnosis of the viral etiology of myocarditis focused on the enteroviruses. More recently, Tavora and collaborators [33] reported a 41-year old African American immunocompetent patient who died of parvoviral myocarditis.

Coxsackievirus B3 (CVB3) is a picornavirus that can also cause viral myocarditis in humans. It has a single-stranded plus sense RNA. The 7.4 kb long genomic RNA is naturally uncapped and contains a viral encoded oligopeptide (VPg), covalently linked at the 5′ end. The highly structured 5′UTR is 741 nt long and has been shown to contain a type I IRES element which helps in mediating internal initiation of translation of CVB3 RNA [34,35]. Type I IRESs occur in PV, CVB3, enterovirus 71 (EV71), and other members of the Enterovirus genus of *Picornaviridae*[6,36]. They are 450 nt long and have 4 major domains (II, IV, V, and VI) but share little homology with type II IRESs except for a Yn-Xm-AUG motif at their 3′ border, in which the AUG triplet is naturally silent [22,36]. Instead, depending on the virus, initiation occurs 30–150 nt downstream of this motif. The mechanism of initiation on type I IRESs has not been solved, and little is known about its requirements for canonical factors or their roles in this process [22].

Internal initiation of translation in CVB3 has been shown to be influenced by various cis-acting elements present in the 5′UTR [37]. The core element of the IRES in CVB3 RNA has been putatively mapped between nt 432–639 of the 5′UTR [38]. This region was shown to possess several critical cis-acting elements which includes a pyrimidine-rich tract and an AUG triplet (AUG^591^) about 25 nucleotides downstream of the pyrimidine-rich tract [37]. It was reported that the MFOLD structure of CVB3 IRES and its secondary structure showed a high degree of similarity to that of poliovirus 5′ UTR [39] and that attenuating mutations for the Sabin vaccine strains of poliovirus are located in domain V; consequently, this domain has received a great deal of experimental interest [40].

Importantly, we have previously addressed the question of whether the attenuating mutations of domain V of the PV IRES were specific for a given genomic context or whether they could be transposed and extrapolated to a genomic related virus, i.e. CVB3. In this context, Ben M’hadheb-Gharbi and collaborators [41,42] have reported that the *Sabin3-like* mutation (U^473^ → C), obtained by direct mutagenesis of the CVB3 genome, and precisely in the domain V sequence of the CVB3 IRES led to a defective mutant with a serious reduction in translation efficiency compared to the wild-type strain. Prediction of the secondary structure by MFOLD program indicated a structural perturbation of the stem containing the *Sabin3-like* mutation, suggesting that specific protein-viral RNA interactions were disrupted, preventing efficient viral translation. The poor translation efficiency of the *Sabin3-like* IRES was then explicated by its inability to correctly bind some essential non-canonical translation factors during the initiation of translation. To fill this gap, we have used a proteomic approach that has allowed the identification of some RNA-binding proteins interacting with both wild-type and mutant CVB3 IRESes [43]. Interestingly, the mutant RNA showed a reduced RNA-protein binding profile [43] and a reduced efficiency of ribosomal complex assembly [44] compared to the wild-type IRES. Taken together, these data were explicated by the inability of the mutant RNA to interact with some trans-acting factors critical for enhanced IRES function. Consequently, we hypothesized that the *Sabin3-like* mutation induces a partial destabilization of the RNA secondary structure of the domain V, leading to a reduced recognition of this region by protein factors necessary for CVB3 translation initiation.

Thus, in order to confirm this hypothesis and with the aim of identifying translation initiation factors specifically binding to domain V of the wild-type and *Sabin3-like* CVB3 RNAs, a proteomic approach was carried out. Over the years, RNA-protein interactions have been determined using different approaches, such as Electrophoretic mobility-shift assays, UV cross-linking or Filter-binding assays. Along this idea, riboproteomic approaches have facilitated the identification of various proteins interacting with different IRES elements. Accordingly, in the present study, we performed UV-crosslinking assays to compare the RNA-protein interaction pattern between CVB3 wild-type and mutant domain V RNAs in the presence of HeLa and BHK21 cell extract proteins. Filter-binding experiments were then carried out in order to assess the binding affinity of some initiation factors: eIF3, eIF4G and eIF4B to both wild-type and *Sabin3-like* domain V RNAs. Interestingly, the mutant RNA showed a reduced protein-binding profile compared to the wild-type domain V. A number of proteins binding to the domain V were identified: p220 (eIF4G), p116 (eIF3) and p80 (eIF4B). Filter-binding assays showed a better binding affinity of eIF3, eIF4G and eIF4B to the wild-type CVB3 domain V. These results perfectly correlate with the impaired protein-binding and the reduced translation efficiency previously reported with the *Sabin3-like* construct [41-44].

## Materials and methods

### Virus

The Coxsackievirus B3 (CVB3) Nancy prototype strain and the *Sabin3-like* mutant of CVB3, used as a “vaccine candidate” and obtained by direct mutagenesis (U^473^ → C) [41] were used for all the experiments. These strains were propaged in Vero cells (African Green Monkey Kidney Cells) (Bio Whittaker) maintained in Eagle’s minimal essential medium supplemented with 10% heat-inactivated fetal calf serum (FCS) (Sigma), 1% L-glutamine, 50 μg/ml de streptomycin, 50 UI/ml de penicillin (Bio Whittaker), 1% non-essential amino acids (Gibco BRL) and 0.05% Fongizone (Amphotericin B, Apothecon).

### Synthesis of the CVB3 domain V by primers- hybridization and extension method

#### Primers design

Based on the sequence of the CVB3 domain V, primers DV_(Wt)_-F (5”- TAT gAA TTC ***TAA TAC gAC TCA CTA TAg*** gTC CTC Cgg CCC CTg AAT gCg gCT AAT CCT AAC TgC ggA gCA CAC ACC CTC AAg CCA gAg ggC AgT gTg TCg TAA -3”)/DV_(*S3*)_-F (5′- TAT gAA TTC ***TAA TAC gAC TCA CTA TAg*** gTC CTC Cgg CCC CTg AAT gCg gCT AAT TCT AAC TgC ggA gCA CAC ACC CTC AAg CCA gAg ggC AgT gTg TCg TAA -3′) and DV-R (5′- TAT ggA TCC ATg AAA CAC ggA CAC CCA AAg TAg TCg gTT CCg CTg CAg AgT TgC CCg TTA CgA CAC ACT gCC CTC Tgg CTT gAg ggT gT -3′) were designed for the synthesis of domain V for both CVB3 wild-type (Wt) and *Sabin3-like* (*S3*) strains by primers- hybridization and extension method. EcoRI restriction site/T7 promoter upstream and BamHI restriction site downstream were introduced into primer’s sequences.

#### Synthesis of the CVB3 wild-type and *Sabin3-like* domains V

Primers- hybridization and extension technique was used as previously described [45] to synthesize the CVB3 domain V (158 nucleotides). This technique consists briefly in reacting a pair of primers amplifying the gene of interest and designed so that an overlapping sequence of about 20 nucleotides between forward and reverse primers is incorporated. The reaction mixture was prepared as follows: Taq Buffer (1X) (Roche), 0.05 mM dNTPs, 100 pmol from each primer DV_(Wt)-_F/DV-R or DV_(*S3*)_-F/DV-R, 1 U Taq polymerase (Roche) and the final volume (50 μL) was adjusted by adding sterile distilled water. The reaction was then incubated in a thermocycler (BioRad) according to the following thermal profile: 4 min at 72°C, 6 min at 44°C and 60 min at 70°C. Amplification products were revealed by electrophoretic migration on 2% agarose gel containing Ethidium Bromide.

### Cloning of the CVB3 domain V

Amplified CVB3 wild-type and mutant domains V were digested with EcoRI and BamHI (Roche Applied Science). Digestion products were, then, purified using the “QIA quick PCR Purification Kit” (Qiagen) and inserted into the pUC19 plasmid (Invitrogen) digested with the appropriate enzymes and purified using the “Wizard SV Gel and PCR Clean-Up system kit” (Promega). Ligation products were, then, transformed in chemocompetent *Escherichia coli* DH5α cells. Transformants were selected in Luria–Bertani (LB) agar supplemented with 100 μg/ml Ampicillin, 64 μg/ml 5-bromo-4-chloro-3-indolyl-beta-D galactopyranoside, and 0.2 mM isopropyl beta-D-thiogalactopyranoside. Blue–white selection was used to identify white clones containing inserts, while blue clones contained undigested cloning vector. White colonies were individually cultured in LB broth.

### PCR-colony and sequencing

In order to analyze the cloned sequences, randomly chosen clones were tested by PCR-colony as previously described [43]. Positive clones were, then, sequenced using an ABI Prism BigDye Terminators Sequencing Kit (Applied Biosystems). Vector primers M13-F (5′-TgT AAA ACg ACg gCC AgT -3′) and M13-R (5′- CAg gAA ACA gCT ATg ACC -3′) (New England Biolabs) were used for sequencing.

### RNA preparation

Plasmids were linearized to obtain transcripts corresponding to wild-type and mutant CVB3 domains V. Following digestion, DNAs were phenol extracted and ethanol precipitated. Transcription was performed for 1 h at 37°C using 50 U of T7 RNA polymerase (New England Biolabs) in the presence of 0.5–1 μg of linearized DNA template, 50 mM DTT, 0.5 mM each rNTP, and 20 U of RNasin (Promega). Transcripts were uniformly labeled to a specific activity of 0.5–1 × 10^6^ cpm/pmol using (α^32^P)-CTP (400 Ci/mmol). Reactions were incubated for 10 min at 37°C with 1 U of RQ1 DNase (Promega), and unincorporated (α^32^P)-CTP was eliminated by exclusion chromatography in TE-equilibrated columns (Microspin G25 columns, Biosciences). RNA was extracted with phenol–chloroform, ethanol precipitated, and resuspended in RNase-free water (1–2 × 10^5^ cpm/μL). Prior to further use, the integrity of probes was verified in 6% acrylamide, 7 M urea denaturing gel. Dried gels were exposed for autoradiography.

### Competitive UV-crosslinking assays

#### Cell-extract preparation

For a comparative analysis of the binding affinity of cellular proteins to domain V of the CVB3 IRES, two cell lines were used: HeLa (Human) and BHK-21 (Hamster) cells, respectively known as prominent and lacking expression of the coxsackie-adenovirus receptor (CAR). These cell lines had been chosen since they represent well established systems for studying viral replication and infectivity. In addition, cells lysates contain host proteins that support internal translation initiation of the viral RNA.

BHK-21 and HeLa cells, grown to 100% confluence in 10 cm dishes in 5% FCS-supplemented DMEM, were washed twice with cold PBS, scraped and collected by centrifugation. The cellular pellet was resuspended in 1–2 volumes of 10 mM HEPES (pH 7.4), 10 mM KAc, 1.5 mM MgAc, and 2.5 mM DTT, and homogenized by 30 strokes in a glass Dounce. Cellular debris was eliminated by centrifugation at 5,000 × *g* for 5 min. The clear lysate was centrifuged at 10,000 × *g* for 5 min, adjusting the supernatant to 3% glycerol [46]. The concentration of protein in the sample was determined by the Bradford assay (Bio-Rad). Total cytoplasmic RNA was isolated from BHK-21 and HeLa cell monolayers, washed twice with cold PBS and lysed in 1 mL of 50 mM Tris–HCl (pH 7.8), 120 mM NaCl, and 0.5% NP40. Following elimination of cellular debris, cytoplasmic RNA was extracted from 200 μl cytosolic extract using TriPure Isolation Reagent (Boehringer Mannheim) as previously described [47]. Following ethanol precipitation, the RNA sample was resuspended in TE (10 mM Tris–HCl pH 8, 1 mM EDTA) and the concentration was estimated from the OD measured at 260 nm.

#### Competitive UV-cross linking

Extracts from HeLa and BHK-21 cells (40 μg of proteins) were incubated with uniformly radiolabeled probes (0.2 pmol, 1–2 9x 10^5^ cpm) in 15 μL of 10 mM HEPES–KOH, pH 7.9, 35 mM KCl, 2 mM MgCl_2_, 10% glycerol, 0.05% NP40, 0.5 mM DTT, 1 μg/μL yeast tRNA (as non-specific competitor). After 15 min of incubation at room temperature, RNA–protein mixtures were exposed to UV light (254 nm, Stratalinker 1800, Stratagene) for 30 min on ice, at 10 cm from the lamp. The non-protected probe in UV-irradiated extracts was digested with RNase A (0.3 μg/μL) during 30 min at 37°C. In parallel, competitive UV-cross-linking assays were performed. Specific competitors (self RNA competitors) were added, five min before adding radioactive probes, at specified molar excess ratios. Yeast tRNA was also used as a non-specific competitor as described above.

The RNA-crosslinked proteins were resolved in 10% SDS–polyacrylamide gel, following addition of the loading buffer, and heating at 90°C for 2 min. When required, an aliquot of crosslinked extracts was treated with proteinase K, in the presence of 0.5% SDS, 30 min at 50°C, followed by 30 min at 37°C. After electrophoresis, gels were dried and the ^32^P-labeled complexes were visualized by autoradiography. Molecular weights of domain V-protein complexes were determined by comparing against a simultaneously loaded protein marker.

### Filter-binding assays

One of the oldest and simplest methods for detecting RNA–protein interactions is the filter-binding assay. If a mixture of RNA and protein is passed through a nitrocellulose filter, the protein is retained and the RNA will pass through. But if the protein is capable of binding RNA, then RNA will be retained on the filter as well. This protocol requires a purified protein of interest and labeled RNA. To perform the assay, the protein sample is serially diluted to several concentrations. It is then mixed with a fixed amount of labeled RNA and allowed to bind under desired conditions for 30–60 min. The binding reactions are then applied to a 96-well dot-blot apparatus with low vacuum to trap the complexes on three membranes: The top membrane traps aggregates, the middle membrane (nitrocellulose) binds proteins and RNA–protein complexes, and the bottom membrane (which is charged) collects free RNA. After washing and drying, the membranes are exposed for quantification.

Accordingly, in the present study, in order to assess the affinity and the specificity of interaction of CVB3 wild-type and *Sabin3-like* domain V RNAs within the initiation factors eIF3, eIF4G and eIF4B, filter-binding assays were carried out.

#### Preparation of initiation factors

Initiation factors (eIF3, eIF4G and eIF4B) were prepared following previously established procedures [13,48].

#### Binding reactions

Labeled RNAs encoding domain V were annealed by heating to 80°C for 2 min in water then cooled at room temperature for 7 min. RNA (10 nM) was then added to a tube containing a folding/binding buffer (20 mM Tris–HCl, 100 mM potassium acetate, 200 mM KCl, 2.5 mM MgCl_2_, 1 mM DTT), and incubated 5 min at room temperature. Initiation factors eIF3, eIF4G and eIF4B were serially diluted immediately before use and then added to reactions. These were incubated at 4°C for 15–30 min before application to the filter. Measurements were performed in parallel within wild-type and mutant domain V RNAs.

#### Filter-binding assays

Filter-binding assays were performed in triplicate using two filters. From top to bottom: a nitrocellulose filter and a charged nylon filter. The filters were pre-soaked in the binding buffer (1X), assembled in a dot blot apparatus or on glass filter funnel and the reactions were applied and directly vacuum filtered. Filters were then rinsed, removed, dried, and radioactivity was quantified using a storm phosphorImager (GE healthcare). The percentage of protein-RNA bound was then calculated and graphs: % binding RNA – protein = f (protein concentration) were drawn.

### Statistical analysis

To assess the binding affinity of CVB3 wild-type and mutant domain V RNAs to eIF3, eIF4G and eIF4B, binding assays were performed and the percentage of protein-RNA bound was calculated. ANOVA test using STATVIEW statistical software package was performed. Normality and homogeneity of data were confirmed before the test and values were considered statistically significant at *p* < 0.05.

## Results

### Cloning of the CVB3 domain V

**Figure 1:**
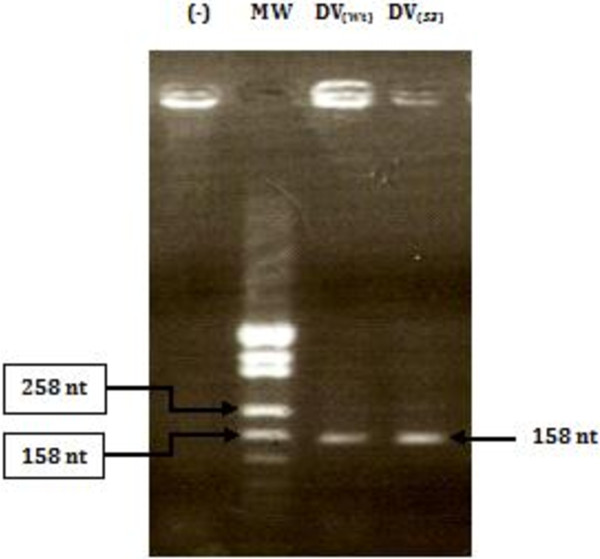
**Electrophoretic profile, observed on 2% agarose gel, of CVB3 wild-type and *Sabin3-like *domain V DNAs**. Lane MW: molecular weight DNA marker; lane (−): a negative control for the reaction; lane DV_(Wt)_: domain V wild-type DNA; lane DV_*(S3)*_: domain V *Sabin3-like* DNA. Experimental conditions were carried out as described in the “Materials and Methods” section.

Two CVB3 strains were analyzed: a wild-type and an attenuated *Sabin3-like* strains. The attenuation of the *Sabin3-like* strain was mainly conferred by a single point mutation in the IRES domain V sequence [41]. Both wild-type and *Sabin3-like* domains V were amplified using the primers- hybridization and extension technique as described in the “Materials and Methods” section. Amplified wild-type and mutant domains V were analyzed on 2% agarose gel (Figure 1). Amplified domain V DNAs (158 nt) were cloned in a pUC19 vector between EcoRI and BamHI restriction sites. Transformed pUC19/IRES clones were confirmed by PCR-colony and sequencing.

### *In vitro* transcription and RNA radioactive labeling

RNAs encoding CVB3 wild-type and *Sabin3-like* domains V were transcribed using the T7 RNA polymerase as described in the “Materials and Methods” section. After transcription, the integrity of these RNAs was verified on an Acryl-Bisacrylamide 6%–7 M Urea gel. Radiolabeled RNAs were then quantified using Biospec-NanoDrop technology.

### Autoradiogram patterns of cellular proteins binding to the CVB3 IRES domain V

According to our previously published data [43,44], we suspected that the *Sabin3-like* mutation would impair the binding of cellular protein factors to the viral RNA that mediate the association of the ribosomal 40S subunit within the CVB3 IRES, and particularly, to the domain V. Four polypeptides of 220, 116, 80 and 57 kDa suspected as eIF4G, eIF3b, eIF4B and PTB, respectively bound to the CVB3 IRES RNA in the presence of BHK-21 cell extract and a reduction in the RNA-protein binding profile for the mutant RNA compared to the wild-type IRES were reported [43].

Interestingly, with the aim of mapping the binding structural sites of initiation factors involved in the CVB3 IRES-dependent translation, we have performed a proteomic approach to identify host factors interacting with domain V of the CVB3 IRES, in parallel to domain V of FMDV as this structure has been previously shown to interact with several identified initiation factors [21,49,50]. To this end, CVB3 wild-type and mutant domain V and FMDV domain V transcripts were incubated with protein extracts prepared from HeLa and BHK-21 cells. A control RNA with unrelated sequence was used to determine the presence of non specific RNA-binding factors, and hence, discard non specific factors from the subsequent analysis. Additionally, to reduce non-specific binding, a large excess of tRNA was added to the incubation mixture.

**Figure 2:**
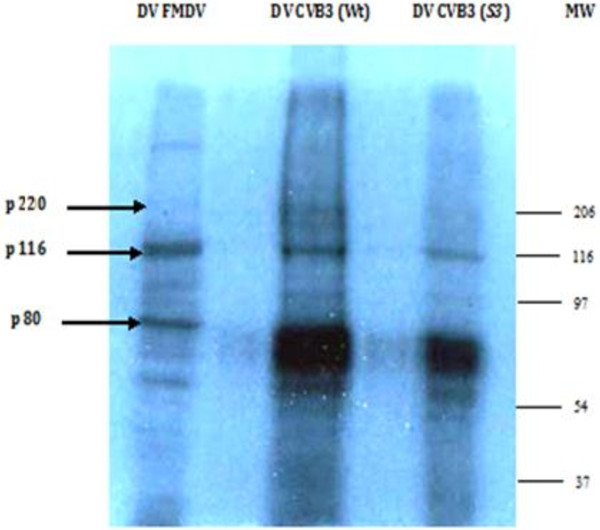
**Autoradiogram of an UV-crosslink assay performed with IRES domain V labeled probes and an extract prepared from HeLa cells.** Proteins were resolved in a 10% SDS-PAGE. Domain V of FMDV IRES was used as positive control (lane DV FMDV). Lanes DV CVB3 _(Wt)_ and DV CVB3 _(*S3*)_ demonstrate the RNA–protein interaction pattern of CVB3 wild-type and *Sabin3-like* IRES domains V. Equal amounts of total proteins (10 μg) were loaded per well. The molecular weight (MW) of each complex was determined by comparing to a concurrently loaded MW marker: Prestained SDS-page standards broad range (Biorad).

**Figure 3:**
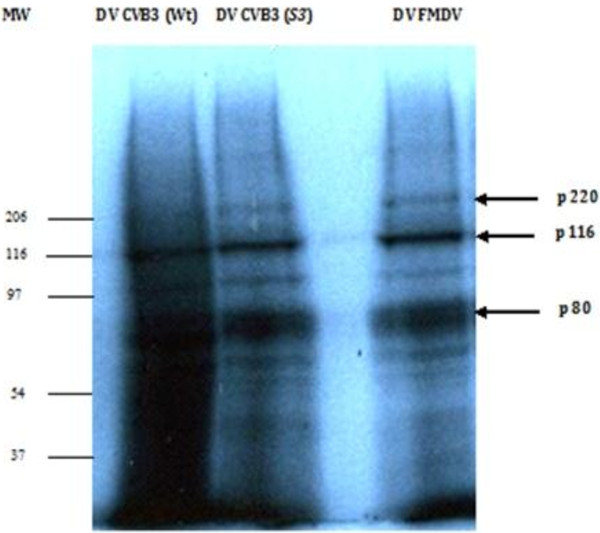
**UV-cross-linking to determine the molecular weight of RNA binding proteins to domain V of the CVB3 IRES**. (α^32^P)-CTP-labeled probes were added to BHK-21 cell total protein extracts and cross-linked by UV-light exposure, followed by RNase A treatment. Proteins that cross-linked to radioactive RNAs were detected by 10% SDS-PAGE and subsequent autoradiography. Lanes DV CVB3 _(Wt)_ and DV CVB3 _*(S3)*_ demonstrate protein-probe interactions within CVB3 wild-type and *Sabin3-like* domain V RNAs, respectively. Lane DV FMDV indicates RNA-protein complexes formed within the FMDV domain V. Equal amounts of total proteins (10 μg) were loaded per well. The molecular weight of each complex was determined by comparing to a concurrently loaded molecular weight (MW) marker: Prestained SDS-page standards broad range (Biorad).

Only proteins that become covalently linked to the RNA upon UV irradiation are revealed by gel autoradiography. The autoradiogram patterns (Figures 2 and 3) showed a reduced interaction of the *Sabin3-like* RNA with several cellular polypeptides compared to the CVB3 wild-type domain V.

In fact, a reduction in the band intensity of *Sabin3-like* domain V-protein complexes was observed; and thus by comparing the intensities of protein-*Sabin3-like* RNA complexes in each gel, we can deduce a relative reduced affinity of the mutant domain V toward cellular proteins present in HeLa and BHK-21 cell extracts. Thus, we can suggest that the *Sabin3-like* mutation leads to a lower efficiency of interaction of the domain V within translation initiation factors present in both cell extracts. As mentioned above, competitive UV-cross-linking assays were performed, in parallel, using a specific (self RNA) and a non-specific (tRNA) competitors for both radiolabeled domain V RNAs (data not shown). Formation of RNA-proteins complexes was reduced by the addition of increasing amounts of unlabeled self RNA. This demonstrates the specificity of the cell protein interactions. In contrast, formation of complexes between cell proteins and yeast tRNA was minimal, as the addition of tRNA did not present significant competition effects.

### CVB3 IRES domain V binds to HeLa and BHK-21 cell extract proteins

In order to assess cellular proteins binding to domain V of the CVB3 IRES, UV- crosslinking assays were performed. Domain V of the FMDV IRES was used as a control probe. The comparative analysis of autoradiogram profiles (Figures 2 and 3) shows that all labeled probes (CVB3 wild-type and mutant, and FMDV RNAs) bound to both cell extracts proteins.

Interestingly, the mutant RNA showed a reduced protein binding profile compared to the wild-type domain V in both cell extracts. To confirm that the reduction of detection of RNA-protein complexes formed within the mutant CVB3 domain V RNA was due to the reduction in the protein binding efficiency to domain V and not to the loss of label transfer from the radioactive RNA to the protein, competitive assays were performed using unlabeled wild-type and *Sabin3-like* RNA competitors. RNAs competed efficiently with the binding of these proteins and to radiolabeled wild-type and mutant RNAs. Thus, the loss of detection of RNA-protein complexes observed with the mutant domain V was due to the loss of protein binding. Taken together, the above results indicate that the domain V contains a major determinant for protein-binding to the CVB3 IRES.

### Determination of molecular weights of the CVB3 domain V- binding proteins

In order to identify proteins that bind to the CVB3 IRES domain V, UV-crosslinking experiments were carried out. RNase A was then added to digest non-interacting regions of the labeled probes, and thus allows realistic molecular weight estimates. Domain V of FMDV IRES was used as a control probe in parallel to CVB3 RNAs. Figures 2 and 3 show some proteins interacting with both FMDV and CVB3 RNAs. We detected a band migrating at an apparent molecular mass of about 116 kDa, which is supposed to be a component of eIF3. Another protein with an apparent molecular mass of about 80 kDa, as expected for eIF4B, was strongly labeled. These 116- and 80- kDa proteins previously identified, respectively, as eIF3 and eIF4B in FMDV [21,49,50] co-migrated with the same mobility for both IRES domain V RNAs suggesting that it could correspond to the same proteins. The identity of these proteins was then confirmed by immunoprecipitation. A third band migrating at an apparent molecular mass of about 220 kDa, as expected for eIF4G [22,43] was also shown to bind, specifically, with CVB3 transcripts. Additionally, several other bands were labeled indicating the presence of other proteins binding to both viral IRES domains V. The smear-like patterns suggest the presence of multiple RNA–protein complexes over a range of molecular weights.

### The Attenuating *Sabin3-like* mutation directly affects the binding of p116 (eIF3), p220 (eIF4G) and p80 (eIF4B) to the CVB3 domain V

Our previous observations that the *Sabin3-like* point mutation in domain V of the CVB3 IRES seriously affected ribosome association [44] and protein-binding [43] within the full IRES sequence, coupled with the fact that domain V is the major determinant for binding of some initiation factors led us to the conclusion that this single nucleotide exchange may directly affect the binding of these translation initiation factors and hence impair the association of the ribosome with the viral RNA. In order to confirm this hypothesis, UV cross-link assays were performed. In the presence of the *Sabin3-like* domain V RNA, the binding of p80 (eIF4B), p116 (eIF3) and p220 (eIF4G) was indeed impaired by this mutation compared to the wild-type RNA; thus correlating with the reduced translation efficiency of the *Sabin3-like* construct [41]. In this experiment, the intensities of bands supposed to be identical to eIF4G, eIF4B and eIF3 were affected by the *Sabin3-like* mutation. Competition experiments confirmed that the observed reduction in band intensities was due to a reduced binding of these factors and not to a reduced label transfer only, while other bands were less strongly affected by the competitions.

### Specific interactions of eIF3, eIF4B and eIF4G with the domain V of the CVB3 IRES

**Figure 4:**
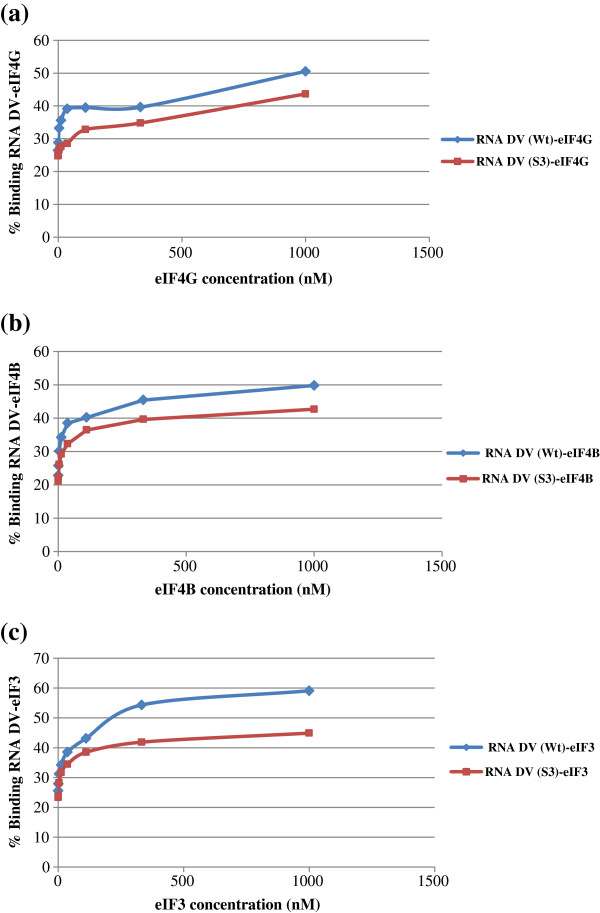
**Filter-binding assays of some initiation factors binding to CVB3 wild-type (Wt) and *Sabin3-like *(S3) Domains V (DV) RNAs**. Purified initiation factors eIF4G **(a)**, eIF4B **(b)**, and eIF3 **(c)** directly bind to the wild-type and the mutant Domain V RNAs.

According to the data obtained by UV-crosslinking experiments, RNA-protein complexes obtained with both CVB3 domain V RNAs were differently susceptible to competitions. The differences in susceptibility to competition can be a consequence of the binding specificities of proteins. Specific interactions were concluded when band intensity corresponds to the decrease in competitor ratios. All proteins appeared to specifically interact with the domain V sequence as they were all susceptible to competitions. To confirm this specificity of RNA–protein interactions, filter-binding assays were carried out. We assayed whether the CVB3 domain V RNA could directly interact with individual initiation factors eIF3, eIF4B and eIF4G. Putative interactions were analyzed by filter-binding assays and the amount of resulting RNA–protein complexes was determined. Values obtained were extrapolated to draw graphs: % binding RNA–protein = f (protein concentration) (Figure 4).

According to ANOVA statistical data, the percentage of RNA protein binding was significantly higher for the CVB3 wild-type RNA (*p* < 0.05) than the *Sabin3-like* RNA for all studied initiation factors; suggesting a better binding affinity of the initiation factors eIF3, eIF4G and eIF4B to the CVB3 wild-type domain V compared with the mutant RNA.

## Discussion

Gene expression control largely depends on ribonucleoprotein complexes regulating mRNA translation. Initiation of translation in mRNAs that overcome cap-dependent translation inhibition is often driven by internal ribosome entry site (IRES) elements, whose activity is regulated by multifunctional RNA binding factors. Understanding the mechanism used by IRES elements to promote internal initiation of translation requires: *(i)* the identification of essential regions in the cis-acting elements (likely involved in the interaction with other regions of the RNA that composes the IRES as well as with cellular proteins), and *(ii)* the identification of translation initiation factors that bring the ribosome into contact with the mRNA. During the last decade, many efforts have been devoted to understand the mechanistic basis of IRES function and how these elements interact with host-cell components in order to recruit the translation machinery [21,28,43,44,49-54].

RNA structure plays a fundamental role in viral IRES dependent translation initiation [1]. In support of this, mutations leading to the disruption of specific RNA structure motifs impaired IRES activity while the corresponding compensatory mutations restored IRES function [9,55]. Furthermore, RNA structure of viral IRES elements is organized in modules which are phylogenetically conserved [56,57], providing evidence in favor of a distribution of functions among the different RNA domains [58,59].

In the best-known example, each of the three attenuated Sabin vaccine strains for poliovirus, considered the prototype picornavirus, contains nucleotide substitutions in domain V of the 5′UTR that are responsible for attenuation [60,61]. These mutants have been shown to multiply poorly in neuronal cells [62], accounting for their decreased neurovirulence and inability to cause poliomyelitis. The attenuating mutations in the Sabin vaccine strains of PV have been identified, and it has been demonstrated that a key determinant of neurovirulence in each of the PV serotypes is located between residues 472 and 481, within domain V of the PV 5′UTR [63]. It has been reported, in the case of poliovirus Sabin vaccine, that even single point mutation in the IRES may change the requirement of the virus ultimately affecting the viral RNA translation [37,64].

Similarly, we have previously reported the limited efficiency of the translation of *Sabin3-like* mutant (U^473^ → C) of CVB3 [41]. Additionally, this *Sabin3-like* point mutation in domain V of the CVB3 IRES seriously affected ribosome association [44] and protein-binding [43] within the full IRES sequence. Thus, the consequence of this mutation was suggested to be a partial destabilization of the RNA secondary structure of the domain V, leading to reduced recognition of this region by protein factors necessary for translation initiation. Consequently, in the present study, in order to identify cellular proteins interacting within the CVB3 domain V, wild-type and mutant domain V transcripts were used in UV-crosslinking assays with *S10* extracts prepared from BHK-21 or HeLa cells that support efficient IRES activity. We have observed a number of proteins binding to the CVB3 domain V RNA. Some of these binding proteins may be identical proteins that have multiple binding sites in the domain V; some of them may be different proteins with similar molecular weights. In fact, three major polypeptides of 220, 116 and 80 kDa, previously identified as eIF4G, eIF3 and eIF4B, respectively, were observed in both cell extracts.

Although molecular weight determinations were not definitive in our UV-cross-linking assays, RNA-protein complexes that we have detected appear to be within reasonable range of these above-mentioned proteins, suggesting possible identities of those CVB3 domain V-binding proteins. To confirm the identity of these proteins, immunoprecipitation assays were carried out. Smear-like patterns represent multiple RNA-protein complexes which could not be distinguished as discrete bands. This suggests that multiple proteins interact with the same RNA molecule and that some of these proteins are in a relative higher content than other specific binding proteins. The RNA–protein interaction pattern could vary depending on the protein source used for the UV-cross-linking assay. This suggests two possible processes: the presence of multiple host proteins that bound the probes at differential affinities and quantities; or the co-operative binding of multiple copies of the same protein toward the probe.

Filter-binding assays results showed a better affinity of binding of the initiation factors eIF3 (p116), eIF4G (p220) and eIF4B (p80) to the wild-type CVB3 IRES domain V compared with the mutant RNA. This finding clearly correlates with the data obtained by UV-cross-linking assays. Taken together, this can be explicated by the inability of the mutant domain V RNA to interact with some trans-acting factors critical for enhanced IRES function. Consequently, here we demonstrate that the reduction in the coxsackievirus B3 translation efficiency caused by the single nucleotide exchange in the IRES domain V sequence of the *Sabin3-like* strain is mediated by an impaired binding of the standard translation initiation factors eIF3, eIF4B and eIF4G to the mutant RNA. This in turn causes impaired association of ribosomes within the viral RNA as was previously reported [44]. In conclusion, the CVB3 IRES domain V is the major determinant for the binding of standard initiation factors eIF3, eIF4G and eIF4B.

Indeed, our present findings correlate with the data published by De Breyne and collaborators [22] who reported that eIF4G specifically recognizes domain V of CVB3, EV71, and PV IRESs, binds to them with the same orientation, and, importantly, does so independently of other factors. These data extend the observation that eIF4G crosslinks to PV IRES domain V in RRL [51]. eIF4G’s binding site is limited to the near-universally-conserved base of domain V. Mutations in this region severely impair translation and consequently impair or even abrogate virus growth [22].

Since there are no more previous reports on protein-RNA interactions of the CVB3, we compare our findings to a close genomic related virus, the poliovirus. Our present results are in a perfect correlation with the previously published data of Ochs and collaborators [51,65] who reported that eIF4B, eIF3, eIF4G, and PTB interact with the poliovirus IRES. They also demonstrated that the reduction in poliovirus translation efficiency caused by single nucleotide exchanges in the IRES of the three poliovirus Sabin vaccine strains is mediated by impaired binding of the standard translation initiation factor eIF4G to the poliovirus IRES domain V and that eIF4G is the crucial factor that initially binds to the poliovirus IRES and recruits the IRES to the other components of the translational apparatus, particularly to the p170 subunit of the ribosome-bound eIF3, thereby mediating association of the viral RNA with the small ribosomal subunit. These findings may have major implications for understanding the attenuation of poliovirus neurovirulence and the pathogenicity of other members of enteroviruses such as CVB3.

Importantly, the introduction of the attenuating Sabin 3 mutation into domain V of the wild-type PV IRES at nucleotide 469 reduced cross-linking of eIF4G and eIF4B to this domain in RRL [51]. These data and the fact that compensatory mutations that restored base-pairing in the base of PV domain V restored IRES-mediated translation and the IRES’s ability to interact with eIF4G strongly support the importance of this interaction for type I IRES function [22].

eIF4G and eIF4A together induced strong toe prints at the 3′ border of type I IRESs (in domain VI and its immediate vicinity) that likely indicate conformational changes in the IRES, because no direct interaction of eIF4G or eIF4A with this region of the IRES was detected [22]. The finding that recruitment of eIF4G/eIF4A to domain V led to these changes in domain VI is consistent with observations suggesting that domains V and VI are functionally linked and might interact structurally, such as their synergy in promoting UV crosslinking to the IRES of a 36-kDa protein in cellular extracts [66] and determining PV neurovirulence [67]. Interestingly, binding of eIF4G/eIF4A to the type II EMCV IRES induced analogous toeprints at its 3′ border [68]. These conformational changes induced at the 3′ borders of type I and II IRESs could be essential for subsequent attachment of 43S complexes to these regions. Although these data strongly suggest that binding of eIF4G/eIF4A is a key step in initiation on type I and II IRESs, it is important to note that it is not sufficient for recruitment of 43S complexes on type II IRESs (and likely also not for type I IRESs) [22].

It was also shown that the binding site for eIF4B in the poliovirus IRES resides mainly in the IRES domain V, whereas domain VI sequences, including the silent AUG at nucleotide 586, contribute to the binding of eIF4B to a lesser extent, which is a finding consistent with the observation that domain VI is not absolutely essential for poliovirus translation [69,70]. In domain V, several linker scanning mutations seriously impaired the binding of eIF4B. These mutations also seriously impaired poliovirus translation and growth, leading to either lethal or temperature-sensitive phenotypes [71]. This correlation suggests that eIF4B may play an important role in the initiation of translation of the Poliovirus. In the relative position of its eIF4B binding site within the arrangement of secondary structures, the poliovirus IRES resembles type II IRES elements like those of FMDV and EMCV [72]. In recombination experiments, the single point mutations in domain V were found to contribute to the attenuation of neurovirulence of the respective Sabin vaccine strains [63] and experiments using chimeras of the poliovirus and rhinovirus IRES elements revealed that the IRES domains V and VI may contain determinants of neuropathogenicity [67].

Probably due to the possible complexity of protein-RNA interactions involved in the activity of the poliovirus IRES, the initiation factor requirements have been investigated so far mainly with the distantly related picornavirus type II IRES elements. The IRES element of FMDV is organized in structural domains, termed 2–5 in 5′- to 3′-end, which appear to have a division of functions [58]. Domains 2, 4 and 5 determine the interaction with RNA binding proteins and various translation initiation factors with the exception of eIF4E [73]. Domains 4 and 5 are responsible for the recruitment of eIF4G, eIF4B, eIF3 and other IRES binding factors [49]. Remarkably, domains 4 and 5 do not possess IRES activity by themselves, indicating that interaction with these factors is necessary but not sufficient for IRES function [50].

eIF4B contains an RNA recognition motif (RRM) near the N-terminus and a second RNA-binding region in the carboxy-terminal half of the protein. Although the carboxy-terminal region binds RNA non specifically with high affinity, the RRM binds such RNAs inefficiently. RNA-binding specificity of the eIF4B RRM has been studied using *in vitro* selection with random sequences. The high affinity ligands showed a conserved set of nucleotides located in a structural motif consisting of a bulge stem-loop [49].

It was reported that eIF3 bound to the FMDV IRES at multiple sites. A preferential eIF3 binding site resided in domain 5. However domain 4 also interacted with this factor and, although to a lower extent, they observed a crosslink of p116 to domains 1–2 and 3. The sequence of conserved residues in domains 4 and 5 is not an essential determinant for eIF3 interaction with FMDV IRES [49]. It remains to be studied whether destabilization of the domain 5 structure impairs eIF3 binding to the FMDV IRES and concomitantly, affects IRES activity.

Extending these results to the type I IRES of poliovirus, Ochs and collaborators [51] showed that eIF4B binds strongly to the poliovirus IRES domain V. Consequently, it can be expected that the basic apparatus of initiation factors acting on the poliovirus IRES is the same as with the EMCV IRES and that eIF4G and eIF4B are directly and functionally involved in the process of translation initiation at the poliovirus and coxsackievirus B3 RNAs.

Although there are similarities among IRES elements in terms of structures and ITAF requirements, there are also specific differences between IRESes even among those of the same classification. The lack of complete conservation of structural elements among viral IRESes and the differences among the viral IRESes in ITAF requirements have made stringent classification difficult. Attending to the essential requirements for internal initiation, IRES elements can be grouped in two main categories: *(i)* those that do not need proteins to assemble the initiation complex and *(ii)* those that do need factors to recruit the ribosome (typically, picornaviruses) [4]. Within the second category, distinct groups can be made depending on the RNA structural motifs and proteins required for activity. Indeed, picornavirus elements IRES belonging to types I and II require the C-terminal end of eIF4G, eIF4A, and eIF3 to assemble 48S initiation complexes [22,49]. Type III IRES require intact eIF4G, and, in contrast, the HCV-like IRES does not need eIF4G to assemble 48S complexes [24].

In addition to eIFs, auxiliary factors termed IRES transacting factors (ITAFs) contribute to modulate (either stimulate or repress) picornavirus IRES activity. In support of the relevance of factors different than eIFs for internal initiation, transcripts encompassing the region interacting with eIFs do not possess IRES activity [4], indicating that interaction with eIFs is necessary but not sufficient for IRES function.

## Conclusions

In summary, the study presented here is an attempt toward the identification of standard initiation factors binding to the domain V of CVB3 IRES and involved in the internal translation initiation of the CVB3 genomic RNA. Interestingly, our present findings implicate that the main effect of the *Sabin3-like* mutation that contributes to *(i)* the attenuation of the cardiovirulence, *(ii)* the reduction efficiency of translation, *(iii)* the impaired ribosomal initiation complex 48S and 80S assembly and (*iv*) the reduction of RNA-protein binding pattern within the full IRES sequence is the impaired binding of the translation initiation factors eIF3, eIF4G and eIF4B to the IRES domain V mutant RNA. Indeed, the *Sabin3-like* mutation could induce a partial destabilization of the secondary structure of domain V, leading to reduced recognition of this region by protein factors necessary for CVB3 translation initiation. This may cause slower translation of the viral RNA and thus may contribute to the attenuated phenotype of the *Sabin3-like* strain.

Nevertheless, the possible involvement of other trans-acting factors (ITAFs) is not ruled out. It can only be speculated if these ITAFs additionally modulate the CVB3 IRES activity, if their binding to *Sabin3-like* mutant is reduced, and if their possibly reduced binding contributes to the attenuated phenotype of the *Sabin3-like* strain. Hence, mapping the exact binding sites of eIF3, eIF4G and eIF4B to domain V of the CVB3 IRES and the identification of other proteins involved in the enhancement of CVB3 IRES-mediated translation would be clearly a challenge in the near future to confirm our results and to increase our understanding of the translation initiation, the 5′UTR-related tissue tropism and the cardiovirulence mechanisms.

Translation initiation mechanisms affecting the efficiency of protein synthesis of a given mRNA are diverse and, importantly, more frequent than anticipated, sometimes giving rise to the expression of different polypeptides from a single transcriptional unit. Therefore, presence of any of these regulatory elements can seriously complicate efforts to accurately define the sites of translation initiation at the genome wide scale.

## Competing interests

The authors declare that they have no competing interests.

## Authors’ contributions

JG and MBMG: participated in the design of the study. AS carried out the experiments and wrote the manuscript. All authors red and approved the final manuscript.

## References

[B1] Martínez-Salas E The impact of RNA structure on picornavirus IRES activity Trends Microbiol 2008 16 230 237 10.1016/j.tim.2008.01.013 18420413 PMC7172834

[B2] Spriggs KA Bushell M Willis AE Translational regulation of gene expression during conditions of cell stress Mol Cell 2010 40 228 237 10.1016/j.molcel.2010.09.028 20965418

[B3] Komar AA Hatzoglou M Cellular IRES-mediated translation: the war of ITAFs in pathophysiological states Cell Cycle 2011 10 229 240 10.4161/cc.10.2.14472 21220943 PMC3048795

[B4] Martìnez-Salas E Piñeiro D Fernàndez N Alternative mechanisms to initiate translation in eukaryotic mRNAs Comp Funct Genomics 2012 2012 2012 10.1155/2012/391546 10.1155/2012/391546 PMC3321441 22536116

[B5] Jang SK Krausslich HG Nicklin MJH Duke GM Palmenberg AC Wimmer E A segment of the 5*′* nontranslated region of encephalomyocarditis virus RNA directs internal entry of ribosomes during *in vitro* translation J Virol 1988 62 2636 2643 2839690 10.1128/jvi.62.8.2636-2643.1988 PMC253694

[B6] Pelletier J Sonenberg N Internal initiation of translation of eukaryotic mRNA directed by a sequence derived from poliovirus RNA Nature 1988 334 320 325 10.1038/334320a0 2839775

[B7] Willcocks MM Locker N Gomwalk Z Structural features of the Seneca valley virus internal ribosome entry site (IRES) element: a picornavirus with a pestivirus-like IRES J Virol 2011 85 4452 4461 10.1128/JVI.01107-10 21325406 PMC3126232

[B8] Yu Y Sweeney TR Kafasla P Jackson RJ Pestova TV Hellen CU The mechanism of translation initiation on aichivirus RNA mediated by a novel type of picornavirus IRES EMBO J 2011 30 4423 4436 10.1038/emboj.2011.306 21873976 PMC3230369

[B9] Honda M Ping LH Rijnbrand RCA Structural requirements for initiation of translation by internal ribosome entry within genome-length hepatitis C virus RNA Virology 1996 222 31 42 10.1006/viro.1996.0395 8806485

[B10] Rijnbrand R van der Straaten T van Rijn PA Spaan WJM Bredenbeek PJ Internal entry of ribosomes is directed by the 5*′* non coding region of classical swine fever virus and is dependent on the presence of an RNA pseudoknot upstream of the initiation codon J Virol 1997 71 451 457 8985370 10.1128/jvi.71.1.451-457.1997 PMC191071

[B11] Vallejos M Ramdohr P Valiente-Echeverría F The 5*′*-untranslated region of the mouse mammary tumor virus mRNA exhibits cap-independent translation initiation Nucleic Acids Res 2009 38 618 632 19889724 10.1093/nar/gkp890 PMC2811009

[B12] Vallejos M Deforges J Plank TD Activity of the human immunodeficiency virus type 1 cell cycle-dependent internal ribosomal entry site is modulated by IRES transacting factors Nucleic Acids Res 2011 39 6186 6200 10.1093/nar/gkr189 21482538 PMC3152342

[B13] Locker N Chamond N Sargueil B A conserved structure within the HIV gag open reading frame that controls translation initiation directly recruits the 40S subunit and eIF3 Nucleic Acids Res 2011 39 2367 2377 10.1093/nar/gkq1118 21071421 PMC3064776

[B14] Czibener C Alvarez D Scodeller E Gamarnik AV Characterization of internal ribosomal entry sites of triatomavirus J Gen Virol 2005 86 2275 2280 10.1099/vir.0.80842-0 16033975

[B15] Lu J Hu Y Hu L Ectropis obliqua picorna-like virus IRES-driven internal initiation of translation in cell systems derived from different origins J Gen Virol 2007 88 2834 2838 10.1099/vir.0.83201-0 17872537

[B16] Fernandez-Miragall O Hernandez C An internal ribosome entry site directs translation of the 3*′*-gene from pelargonium flower break virus genomic RNA: implications for infectivity PLoS ONE 2011 6 e22617 10.1371/journal.pone.0022617 21818349 PMC3144232

[B17] Garlapati S Wang CC Structural elements in the 5*′*-untranslated region of giardiavirus transcript essential for internal ribosome entry site-mediated translation initiation Eukaryot Cell 2005 4 742 754 10.1128/EC.4.4.742-754.2005 15821134 PMC1087810

[B18] Isaksson A Berggren M Ekeland-Sjoberg K Samuelsson T Ricksten A Cell specific internal translation efficiency of Epstein-Barr virus present in solid organ transplant patients J Med Virol 2007 79 573 581 10.1002/jmv.20854 17385682

[B19] Merrick WC Cap-dependent and cap-independent translation in eukaryotic systems Gene 2004 332 1 11 15145049 10.1016/j.gene.2004.02.051

[B20] Sonenberg N Hinnebusch AG Regulation of translation initiation in eukaryotes: mechanisms and biological targets Cell 2009 136 731 745 10.1016/j.cell.2009.01.042 19239892 PMC3610329

[B21] Pacheco A Reigadas S Martínez-Salas E Riboproteomic analysis of polypeptides interacting with the internal ribosome-entry site element of foot-and-mouth disease viral RNA Proteomics 2008 8 4782 4790 10.1002/pmic.200800338 18937254

[B22] De Breyne S Yu Y Unbehaun A Pestova TV Hellen CUT Direct functional interaction of initiation factor eIF4G with type 1 internal ribosomal entry sites Proc Natl Acad Sci U S A 2009 106 9197 9202 10.1073/pnas.0900153106 19470487 PMC2695064

[B23] Belsham GJ Divergent picornavirus IRES elements Virus Res 2009 139 183 192 10.1016/j.virusres.2008.07.001 18675861

[B24] Lukavsky PJ Structure and function of HCV IRES domains Virus Res 2009 139 166 171 10.1016/j.virusres.2008.06.004 18638512 PMC2726286

[B25] Pacheco A Martinez-Salas E Insights into the biology of IRES elements through riboproteomic approaches J Biomed Biotechnol 2010 2010 458927 20150968 10.1155/2010/458927 PMC2817807

[B26] Fernandez-Miragall O Martinez-Salas E Structural organization of a viral IRES depends on the integrity of the GNRA motif RNA 2003 9 1333 1344 10.1261/rna.5950603 14561883 PMC1287055

[B27] Berry KE Waghray S Mortimer SA Bai Y Doudna JA Crystal structure of the HCV IRES central domain reveals strategy for start-codon positioning Structure 2011 19 1456 1466 10.1016/j.str.2011.08.002 22000514 PMC3209822

[B28] Fernandez N Garcia-Sacristan A Ramajo J Briones C Martinez-Salas E Structural analysis provides insights into the modular organization of picornavirus IRES Virology 2011 409 251 261 10.1016/j.virol.2010.10.013 21056890

[B29] Nakashima N Uchiumi T Functional analysis of structural motifs in dicistroviruses Virus Res 2009 139 137 147 10.1016/j.virusres.2008.06.006 18621089

[B30] Lee EK Kim W Tominaga K Martindale JL Yang X Subaran SS Carlson OD Mercken EM Kulkarni RN Akamatsu W RNA-binding protein HuD controls insulin translation Mol Cell 2012 45 826 835 10.1016/j.molcel.2012.01.016 22387028 PMC3319250

[B31] Sweet T Kovalak C Coller J The DEAD-Box protein Dhh1 promotes decapping by slowing ribosome movement PLoS Biol 2012 10 e1001342 10.1371/journal.pbio.1001342 22719226 PMC3373615

[B32] Lakhan SE Harle L Cardiac fibrosis in the elderly, normotensive athlete: case report and review of the literature Diagn Pathol 2008 3 12 10.1186/1746-1596-3-12 18353184 PMC2277381

[B33] Tavora F Gonzalez-Cuyar LF Dalal JS O’Malley MT Zhao R Peng HQ Burke AP Fatal parvoviral myocarditis: a case report and review of literature Diagn Pathol 2008 3 21 10.1186/1746-1596-3-21 18447927 PMC2396599

[B34] Yang D Wilson JE Anderson DR Bohunek L Cordeiro C Kandol R Mcmanus BM *In vitro* mutational and inhibitory analysis of the *cis*-actingtranslational elements within the 5′ untranslated region of coxsackievirus B3: potential targets for antiviral action of antisense oligomers Virology 1997 228 63 73 10.1006/viro.1996.8366 9024810

[B35] Yang D Cheung P Sun Y Yuan J Zhang H Carthy CM Anderson DR Bohunek L Wilson JE Mcmanus BM A shine-dalgarno-like sequence mediates *in vitro* ribosomal internal entry and subsequent scanning for translation initiation of coxsackievirus B3 RNA Virology 2003 305 31 43 10.1006/viro.2002.1770 12504538

[B36] Jackson RJ Kaminski A Internal initiation of translation in eukaryotes: the picornavirus paradigm and beyond RNA 1995 1 985 1000 8595564 PMC1369335

[B37] Bhattacharyya S Verma B Pandey G Das S The structure and function of a cis-acting element located upstream of the IRES that influences coxsackievirus B3 RNA translation Virology 2008 377 345 354 10.1016/j.virol.2008.04.019 18533219

[B38] Liu Z Carthy CM Cheung P Bohunek L Wilson JE McManus BM Yang D Structural and functional analysis of the 5′ untranslated region of coxsackievirus B3 RNA: *In vivo* translational and infectivity studies of full-length mutants Virology 1999 265 206 217 10.1006/viro.1999.0048 10600593

[B39] Skinner MA Racaniello VR Dunn G Cooper J Minor PD Almond JW New model for the secondary structure of the 5′ non-coding RNA of poliovirus is supported by biochemical and genetic data that also show that RNA secondary structure is important in neurovirulence J Mol Biol 1989 207 379 392 10.1016/0022-2836(89)90261-1 2547075

[B40] Hunziker IP Cornell CT Whitton JL Deletions within the 5′UTR of coxsackievirus B3: consequences for virus translation and replication Virology 2007 360 120 128 10.1016/j.virol.2006.09.041 17084431 PMC2190293

[B41] Ben M’hadheb-Gharbi M Gharbi J Paulous S Brocard M Komaromva A Aouni M Kean KM Effects of the *Sabin-like* mutations in domain V of the internal ribosome entry segment on translational efficiency of the coxsackievirus B3 Mol Genet Genomics 2006 276 402 412 10.1007/s00438-006-0155-3 16909284

[B42] Ben M’hadheb-Gharbi M Kean KM Gharbi J Molecular analysis of the role of IRES stem-loop V in replicative capacities and translation efficiencies of coxsackievirus B3 mutants Mol Biol Rep 2009 36 255 262 10.1007/s11033-007-9174-3 18027104

[B43] Souii A Ben M’hadheb-Gharbi M Aouni M Gharbi J *In vitro* molecular characterization of RNA–proteins interactions during initiation of translation of a wild-type and a mutant coxsackievirus B3 RNAs Mol Biotechnol2013a 54 515 527 10.1007/s12033-012-9592-x 22923320

[B44] Souii A Ben M’hadheb-Gharbi M Sargueil B Brossard A Chamond N Aouni M Gharbi J Ribosomal initiation complex assembly within the wild-strain of coxsackievirus B3 and live-attenuated *Sabin3-like* IRESes during the initiation of translation Int J Mol Sci2013b 14 4400 4418 10.3390/ijms14034400 23439549 PMC3634407

[B45] Souii A Gharbi J Ben M’hadheb-Gharbi M Molecular analysis of RNA-RNA interactions between 5′ and 3′ untranslated regions during the initiation of translation of a cardiovirulent and a live-attenuated coxsackievirus B3 strains Int J Mol Sci2013c 14 4525 4544 10.3390/ijms14034525 23439556 PMC3634434

[B46] Lòpez de Quinto S Martìnez-Salas E Interaction of the eIF4G initiation factor with the aphthovirus IRES is essential for internal initiation of translation *in vivo* RNA 2000 6 1380 1392 10.1017/S1355838200000753 11073214 PMC1370009

[B47] Lòpez de Quinto S Saiz M de la Morena D Sobrino F Martinez-Salas E IRES-driven translation is stimulated separately by the FMDV 3′-NCR and poly (A) sequences Nucleic Acids Res 2002 30 4398 4405 10.1093/nar/gkf569 12384586 PMC137133

[B48] Pisarev AV Unbehaun A Hellen CU Pestova TV Assembly and analysis of eukaryotic translation initiation complexes Methods Enzymol 2007 430 147 177 17913638 10.1016/S0076-6879(07)30007-4

[B49] Lòpez de Quinto S Lafuente E Martìnez-Salas E IRES interaction with translation initiation factors: functional characterization of novel RNA contacts with eIF3, eIF4B, and eIF4GII RNA 2001 7 1213 1226 10.1017/S1355838201010433 11565745 PMC1370167

[B50] Fernández N Fernandez-Miragall O Ramajo J García-Sacristá A Bellora N Eyras E Briones C Martínez-Salas E Structural basis for the biological relevance of the invariant apical stem in IRES-mediated translation Nucleic Acids Res 2011 39 8572 8585 10.1093/nar/gkr560 21742761 PMC3201876

[B51] Ochs K Zeller A Saleh L Bassili G Song Y Sonntag A Niepmann M Impaired binding of standard initiation factors mediates poliovirus translation attenuation J Virol 2003 77 115 122 10.1128/JVI.77.1.115-122.2003 12477816 PMC140626

[B52] Fraser CS Doudna JA Structural and mechanistic insights into hepatitis C viral translation initiation Nature Rev Microbiol 2007 5 29 38 10.1038/nrmicro1558 17128284

[B53] Fitzgerald KD Semler BL Bridging IRES elements in mRNAs to the eukaryotic translation apparatus Biochim Biophys Acta 2009 1789 518 528 10.1016/j.bbagrm.2009.07.004 19631772 PMC2783899

[B54] Verma B Bhattacharyya S Das S Polypyrimidine tract binding protein interacts with coxsackievirus B3 RNA and influences its translation J Gen Virol 2010 91 1245 1255 10.1099/vir.0.018507-0 20071487

[B55] Martínez-Salas E Regalado MP Domingo E Identification of an essential region for internal initiation of translation in the aphthovirus internal ribosome entry site and implications for viral evolution J Virol 1996 70 992 998 8551640 10.1128/jvi.70.2.992-998.1996 PMC189904

[B56] Martínez-Salas E Fernandez-Miragall O Picornavirus IRES: structure-function relationship Curr Pharm Design 2004 10 3757 3767 10.2174/1381612043382657 15579069

[B57] Barria MI Gonzalez A Vera-Otarola J Analysis of natural variants of the hepatitis C virus internal ribosome entry site reveals that primary sequence plays a key role in cap-independent translation Nucleic Acids Res 2009 37 957 971 10.1093/nar/gkn1022 19106142 PMC2647302

[B58] Serrano P Ramajo J Martínez-Salas E Rescue of internal initiation of translation by RNA complementation provides evidence for a distribution of functions between individual IRES domains Virology 2009 388 221 229 10.1016/j.virol.2009.03.021 19383564

[B59] Jang CJ Jan E Modular domains of the dicistroviridae intergenic internal ribosome entry site RNA 2010 16 1182 1195 10.1261/rna.2044610 20423979 PMC2874170

[B60] Kawamura NM Kohara M Abe S Komatsu T Tago K Arita M Nomoto A Determinants in the 5′ non coding region of poliovirus Sabin 1 RNA that influence the attenuation phenotype J Virol 1989 63 1302 1309 2536835 10.1128/jvi.63.3.1302-1309.1989 PMC247827

[B61] Macadam AJ Pollard SR Ferguson G Dunn G Skuce R Almond J Minor PD The 5′ non coding region of the type 2 poliovirus vaccine strain contains determinants of attenuation and temperature sensitivity Virology 1991 181 451 458 10.1016/0042-6822(91)90877-E 1707566

[B62] La Monica N Racaniello VR Differences in replication of attenuated and neurovirulent polioviruses in human neuroblastoma cell line SH-SY5Y J Virol 1989 63 2357 2360 2539524 10.1128/jvi.63.5.2357-2360.1989 PMC250657

[B63] Ren R Moss EG Racaniello VR Identification of two determinants that attenuate vaccine-related type-2 poliovirus J Virol 1991 65 1377 1382 1847458 10.1128/jvi.65.3.1377-1382.1991 PMC239915

[B64] Guest S Pilipenko E Sharma K Chumakov K Roos RP Molecular mechanisms of attenuation of the Sabin strain of poliovirus type 3 J Virol 2004 78 11097 11107 10.1128/JVI.78.20.11097-11107.2004 15452230 PMC521805

[B65] Ochs K Saleh L Bassili G Sonntag VH Zeller A Niepmann M Interaction of translation initiation factor eIF4B with the poliovirus internal ribosome entry site J Virol 2002 76 2113 2122 10.1128/jvi.76.5.2113-2122.2002 11836388 PMC135939

[B66] Haller AA Semler BL Stem-loop structure synergy in binding cellular proteins to the 5′ non coding region of poliovirus RNA Virology 1995 206 923 934 10.1006/viro.1995.1015 7856105

[B67] Gromeier M Bossert B Arita M Nomoto A Wimmer E Dual stem loops within the poliovirus internal ribosomal entry site control neurovirulence J Virology 1999 73 958 964 9882296 10.1128/jvi.73.2.958-964.1999 PMC103915

[B68] Kolupaeva VG Lomakin IB Pestova TV Hellen CU Eukaryotic initiation factors 4G and 4A mediate conformational changes downstream of the initiation codon of the encephalomyocarditis virus internal ribosomal entry site Mol Cell Biol 2003 23 687 698 10.1128/MCB.23.2.687-698.2003 12509466 PMC151537

[B69] Kuge S Nomoto A Construction of viable deletion and insertion mutants of the Sabin strain type 1 poliovirus: function of the 5′ non coding sequence in viral replication J Virol 1987 61 1478 1487 3033275 10.1128/jvi.61.5.1478-1487.1987 PMC254126

[B70] Meerovitch K Nicholson R Sonenberg N *In vitro* mutational analysis of cis-acting RNA translational elements within the poliovirus type 2 5′ untranslated region J Virol 1991 65 5895 5901 1656078 10.1128/jvi.65.11.5895-5901.1991 PMC250252

[B71] Haller AA Semler BL Linker scanning mutagenesis of the internal ribosome entry site of poliovirus RNA J Virol 1992 66 5075 5086 1321289 10.1128/jvi.66.8.5075-5086.1992 PMC241370

[B72] Niepmann M Internal translation initiation of picornaviruses and hepatitis C virus Biochim Biophys Acta 2009 1789 529 541 10.1016/j.bbagrm.2009.05.002 19439208

[B73] Martínez-Salas E Pacheco A Serrano P Fernandez N New insights into internal ribosome entry site elements relevant for viral gene expression J Gen Virol 2008 89 611 626 10.1099/vir.0.83426-0 18272751

